# Comparison in antioxidant and antitumor activities of pine polyphenols and its seven biotransformation extracts by fungi

**DOI:** 10.7717/peerj.3264

**Published:** 2017-05-23

**Authors:** Hui Li, Zhenyu Wang

**Affiliations:** School of Chemistry and Chemical Engineering, Harbin Institute of Technology, Harbin, Heilongjiang, China

**Keywords:** Biotransformation, Polyphenols, Antioxidant, *Aspergillus*, Antitumor

## Abstract

Microbial transformation can strengthen the antioxidant and antitumor activities of polyphenols. Polyphenols contents, antioxidant and antitumor activities of pine polyphenols and its biotransformation extracts by *Aspergillus niger*, *Aspergillus oryzae*, *Aspergillus carbonarius*, *Aspergillus candidus*, *Trichodermas viride*, Mucor wutungkiao* and Rhizopus sp* were studied. Significant differences were noted in antioxidant and antitumor activities. The highest antioxidant activities in Trolox equivalent antioxidant capacity (TEAC), DPPH radical scavenging activity, superoxide anion radical scavenging activity, hydroxyl radical scavenging activity, reducing power assay and antitumor activity against LoVo cells were biotransformation extract of *Aspergillus carbonarius* (BAC), biotransformation extract of *Mucor wutungkiao* (BMW), biotransformation extract of *Aspergillus carbonarius* (BAC), biotransformation extract of *Aspergillus niger* (BAN), biotransformation extract of *Aspergillus oryzae* (BAO) and BMW, respectively. Correlation analysis found that antioxidant and antitumor activities were associated with polyphenols contents and types of free radicals and tumors. *A. carbonarius* can make polyphenol oxidation, hydroxylation and methylation, and form new polyphenols. In conclusion, *A. carbonarius*, *A. niger and M. wutungkiao* are valuable microorganisms used for polyphenols biotransformation and enhance the antioxidant and antitumor activities of polyphenols.

## Introduction

The main ingredient of pine barks extracts is proanthocyanidins, followed by catechin-based flavonoids and phenolic acids. These proanthocyanidins and catechin-based flavonoids have high potential values of physiological activities, such as antioxidants (Grimm, Schäfer & Högger, 2004), anti-tumors (Ma et al., 2008), anti-inflammatory (Peng et al., 2012), anti-neurodegenerative diseases (Pipingas et al., 2008), anti-atherosclerosis (Kim et al., 2010), anti-UV radiation (Kimura & Sumiyoshi, 2010) and anti-radiation (Li et al., 2016), etc.

The natural polyphenols have definite limitations in applications because of some shortcomings such as low bioavailability. The *β*-glucosidase-producing fungi including *Aspergillus niger, Aspergillus niveus* and *Aspergillus awamori* can enhance the *in vitro* and *in vivo* antioxidant activities of fermented methanolic extracts and increase the content of free polyphenols of soybean flours (Georgetti et al., 2009). *Streptococcus thermophilus* and *Lactobacillus casei*-01 biotransformation is a feasible and efficient method to convert litchi pericarp proanthocyanidins to a more effective antioxidant agent (Li et al., 2013). The tannase from *Paecilomyces variotti* is able to modify the polyphenol composition of orange juice, act in naringin and hesperidin for the removal of glycosides, and heighten functional activity in relation to the original samples, as demonstrated by *in vitro* tests of antioxidant activities (ORAC and DPPH) and an antiproliferative assay of anthropogenic tumor cells (Ferreira et al., 2013). The bioavailability of chlorogenic acid depends largely on its metabolism by the gut microbes (Gonthier et al., 2003). *Pichia kudriavzevii* ZJPH0802 can transform curcumin into hexahydrocurcumin and tetrahydrocurcumin, and enhance its solubility (Zhang et al., 2013). *Aspergillus terreus* can transform rutin into isoquercitrin with strong anti-inflammatory activity (Weignerova et al., 2012).

Microorganisms for biotransformation mainly originate in fermented foods, such as fermented soy, wine and yoghurt. Lactic acid bacteria, bacillus, yeast and aspergillums are mainly representative microorganisms (Hur et al., 2014). *Aspergillus niger* can decompose valonea tannins into ellagic acid (Shi et al., 2005). The *Aspergillus awamori* fermentation of litchi pericarps generates quercetin, kaempferol and their glycoside derivatives (Lin et al., 2014a; Lin et al., 2014b). However, fungal biotransformation of the pine polyphenols is rarely reported. In this study, antioxidant and antitumor activities *in vitro* against anthropogenic tumor cells of the pine polyphenols (PPs) and its biotransformation extracts by *Aspergillus niger* (BAN), *Aspergillus oryzae* (BAO), *Aspergillus carbonarius* (BAC), *Aspergillus  candidus* (BACS), *Trichodermas viride* (BTV), *Mucor wutungkiao* (BMW) and *Rhizopus sp* (BRS) are compared. The aim of this study is to prove beneficial fungi in enhancing the polyphenols antioxidant and antitumor activities *in vitro*.

## Materials and Methods

### Reagents and chemicals

6-Hydroxy-2,5,7,8-tetramethylchroman-2-carboxylic acid (Trolox), Phenazine methosulphate (PMS), 3-(4,4-Dimethylthiazol-2-yl)-2,5-diphenyltetrazolium bromide (MTT), 2, 2′-Azino-bis(3-ethylbenzothiazoline-6-sulfonic acid) diammonium salt (ABTS), Nitrotetrazolium blue chloride (NBT) and 2,2-Diphenyl-1-picrylhydrazyl (DPPH) were purchased from Sigma (St. Louis, MO, USA); Nicotinamide adenine dinucleotide (NADH) was purchased from Roche, Switzerland; 1 M Folin-Ciocalteu reagent was obtained from Tianjin Guangfu Fine Chemical Research Institute (Tianjin, China); D101 macroporous resins were obtained from the Chemical Plant of Nankai University (Tianjin, China); Food grade 95% ethanol was purchased from a local reagents corporation.

#### Preparation of pine polyphenols

Because of annual and renewable availability, pine cones (without pine nuts) were applied in the study. Pine polyphenols (PPs) were perpetrated according to our previous method (Li & Wang, 2015). In order to increase the purity of polyphenols, the chromatographic column of D101 macroporous resins was used to enrich polyphenols again. 40% (v/v) ethanol solution was used to elute polyphenols. Finally, a 57.7% purity of polyphenols was achieved.

#### Strains used

*Aspergillus niger, Aspergillus oryzae, Aspergillus carbonarius, Aspergillus candidus, Trichodermas viride, Mucor wutungkiao* and *Rhizopus sp* were preserved in our laboratory. Fungal strains were inoculated on Potato Dextrose Agar (PDA), composed of (g/L) peeled potatoes 200, dextrose 20, and agar 15, incubated at 28 °C in darkness. To obtain pure cultures, mycelium was repeatedly transferred onto plates with Basal Medium (BM, g/L) (Contreras-Domínguez et al., 2006) composed of dextrose 20, (NH_4_)_2_SO_4_ 3, KH_2_PO_4_ 1.3, Na_2_HPO4 0.12, MgSO_4_⋅5H_2_O 0.3, CaCl_2_ 0.02 and agar 15.

#### Biotransformation conditions and products enriched

The liquid medium employed in biotransformation was BM broth, tween-80 (0.1%, v/v) and trace elements (1 mL/L). Trace elements are composed of (g/L) FeSO_4_⋅7H_2_O 5, ZnSO_4_⋅7H_2_O 1.4, MnSO_4_⋅H_2_O 1.6, CuSO_4_⋅5H_2_O 0.3 and CoCl_2_⋅6H_2_O 3.7, which were adjusted to pH 6.0 with 6 N HCl before autoclaving. Media was sterilized by autoclaving at 121 °C for 20 min. Microorganisms were cultivated in two stages in a medium (Sanchez-Gonzalez & Rosazza, 2006). The first phase cultivation was used to start the second phase. In first phase, 10^7^ fungal spores were administered to 500 mL conical flasks with 200 mL liquid media, and grew for 48 h at 28 °C, 150 rpm in a SHA-C incubator (Jiangsu, China). Then, in the second phase, 10 mL of concentrated sterile polyphenols solution was added to cultivation to a final medium concentration of 0.4 mg/mL, and continued to cultivate for 48 h at 28 °C, 150 rpm. Finally, filter paper was used to filter medium to remove mycelia. The final liquid was the biotransformation liquid. Biotransformation liquid or cultures without polyphenols was loaded into the D101 macroporous resin chromatographic column; 60% (v/v) ethanol solution eluted products. The elution of 2 BV was gathered and concentrated by removing the ethanol using a rotary evaporator device (RE52AA; Shanghai Huxi Instrument Co., Changsha, China) at 50 °C. Finally, biotransformation extracts were freeze-dried by vacuum (Zirbus, Bad Grund, Germany) in −50 °C  for 24–48 h.

#### Determination of polyphenols content

Polyphenols content of the PPs and seven biotransformation extracts was determined by Folin-Ciocalteu method with some modifications (Lin et al., 2012a; Lin et al., 2012b). Sample solution of 0.5 mL was transferred to a 10 mL test tube, to which 0.5 mL of 1 M Folin-Ciocalteu reagent was added. After 5 min, 1.0 mL of 10% (w/v) Na_2_CO_3_ was added and the volume was added to 3 ml with deionized water. After 2 h of incubation at room temperature, the absorbance was measured at 760 nm in a SPECORD^®^200 Plus UV/VIS Spectrophotometers. Water (for PPs) or cultures without polyphenols (for biotransformation extract, respectively) were used the blank. Polyphenols content was then expressed as mg of gallic acid equivalents/mg of samples. The regression line was *y* = 0.01235*x* + 0.01302 (*R*^2^ = 0.9994, *n* = 6), where *y* is OD_760nm_ of gallic acid and *x* is gallic acid concentration (0–50 µg/mL).

#### Determination of yield of polyphenols in biotransformation extract

According to the following formula, yield of polyphenols in seven biotransformation extracts was calculated.

*Y*  = Polyphenols content in biotransformation extracts/Polyphenols content before biotransformation 100%.

Where *Y* is yield of polyphenols in biotransformation extract.

### Antioxidant activity

#### Trolox equivalent antioxidant capacity (TEAC)

Trolox equivalent antioxidant capacity of the PPs and seven biotransformation extracts was carried out (Wang et al., 2013). Briefly, an ABTS solution (7 mM) was mixed with potassium persulfate (140 mM) with a ratio of 62.5:1 for 16 h in darkness at room temperature to produce ABTS radical cation (ABTS^⋅+^) stock solutions. The ABTS^⋅+^ stock solution was diluted to an absorbance of 0.70 ± 0.05 at 734 nm as a working solution. An aliquot (5 µL) of aqueous solution containing 1 mg/mL sample was mixed thoroughly with the ABTS^⋅+^ solution (200 µL) and after 6 min in darkness at room temperature; the absorbance was read at 734 nm in a gene 5 microplate reader. Different levels (0, 7.8125, 15.625, 31.25, 62.5, 125, 250, and 500 µg/mL) of Trolox standard solutions were prepared and assayed under the same condition. Water (for PPs) or cultures without polyphenols (for biotransformation extract, respectively) were used the blank. Results were taken into account in Trolox equivalent, i.e., µg Trolox/mg samples. The regression line was *y* = 0.0853*x* − 0.3306 (*R*^2^ = 0.9992, *n* = 6), where *y* is ABTS^⋅+^ radical scavenging activity and *x* is Trolox concentration (µg/mL).

#### DPPH free radical scavenging activity

DPPH free radical scavenging activity of the PPs and seven biotransformation extracts was measured by means of the absorbance of DPPH at 517 nm (Xia et al., 2014). Briefly, 1.0 mL sample solution was added to 1.0 mL of DPPH solution (0.2 mM in methanol). The decrease in the solution absorbance at 517 nm was measured after 30 min of incubation. Water (for PPs) or cultures without polyphenols (for biotransformation extract, respectively) were used the blank. DPPH free radical scavenging activity was calculated using the following formula:

\begin{eqnarray*}\text{Scavenging activity}(\text{%})=(1-{A}_{1}/{A}_{0})\times 100 \end{eqnarray*}

where *A*_0_ is the absorbance of DPPH free radicals without sample, and *A*_1_ is the absorbance of DPPH free radicals with samples. The efficient concentration of samples that scavenged 50% of DPPH free radical (EC_50_) was calculated and expressed as µg/mL.

#### Superoxide anion radical scavenging activity

The superoxide anion radical scavenging activity of the PPs and seven biotransformation extracts was established by monitoring the competition of those with NBT for the superoxide anion generated by the PMS–NADH system (Biswas, Chatli & Sahoo, 2012). The reduction mixture contained 150 µL NBT (100 µM), 450 µL NADH (100 µM) and a sample solution 200 µL. Total volume was made up to 1 mL with distilled water and then 1.9 mL of Tris–HCl buffer (0.02 M, pH 8.0) was added. The reaction was started by adding 100 µL of PMS (100 µM) and then the change in absorbance (A) was recorded at 560 nm after 1 min at 37 °C. Water (for PPs) or cultures without polyphenols (for biotransformation extract, respectively) were used the blank. The superoxide anion free radical scavenging activity was calculated with the following equation:

\begin{eqnarray*}\text{Scavenging activity}(\text{%})=(1-{A}_{1}/{A}_{0})\times 100 \end{eqnarray*}

where *A*_0_ is the absorbance of superoxide anion radicals without sample; and *A*_1_ is the absorbance of superoxide anion radicals with sample. The efficient concentration of samples that scavenged 50% of the superoxide anion radicals (EC_50_) was calculated and expressed as µg/mL.

#### Hydroxyl radical scavenging activity

The hydroxyl radical scavenging activity of the PPs and seven biotransformation extracts was investigated using Fenton’s reaction (Fe^2+^ + H_2_O_2_ → Fe^3+^ + OH −  + OH^−^) (Jiang, Chen & Shi, 2013). The reaction mixture containing 1 mL sample solution was incubated at 37 °C  for 2 h with 1 mL of 9 mM salicylic acids, 1 mL of 9 mM FeSO_4_, and 1 mL of 8.8 mM H_2_O_2_, and then the absorbance was read at 510 nm. Water (for PPs) or cultures without polyphenols (for biotransformation extract, respectively) were used the blank. The hydroxyl radical scavenging activity was calculated from the following equation:

\begin{eqnarray*}\text{Scavenging activity %}=[1-({A}_{\mathrm{i}}-{A}_{\mathrm{j}})/{A}_{0}]\times 100 \end{eqnarray*}

where *A*_0_ is the absorbance of the hydroxyl radical with a treated control. *A*_i_ and *A*_j_ are the absorbances of the hydroxyl radical with the treated sample, and the absorbance of the hydroxyl radical with the non-treated sample. The efficient concentration of samples that scavenged 50% of the hydroxyl radicals (EC_50_) was calculated and expressed as µg/mL.

#### Reducing power (RP) assay

The RP assay of the PPs and seven biotransformation extracts was conducted (Seo et al., 2012). A freshly prepared sodium phosphate buffer (1.0 mL, 0.2 M, pH 6.6) and 1% K_3_Fe (CN)_6_ (1.0 mL) were added to the sample solution (1.0 mL). After incubating the mixtures at 50 °C for 20 min, 10% trichloroacetic acid (1.0 mL) was added. The resulting mixtures were centrifuged at 3,000 rpm for 10 min at 4 °C. The upper layer (1.0 mL) was diluted with water (1.0 mL), and 0.1 % FeCl_3_ (0.5 mL) was hereby added. The absorbance of the resultant solution was measured at 700 nm. Water (for PPs) or cultures without polyphenols (for biotransformation extract, respectively) were used the blank. The efficient concentration of samples that were of 0.5 absorbance value (RP_0.5_) was calculated and expressed as µg/mL.

### Antitumor activity

#### Cell lines and culture conditions

LoVo (human colon adenocarcinoma cell), HeLa 60 (human cervical carcinoma cell) and BxPC-3 (human pancreatic carcinoma cell) cells were preserved in our laboratory and were cultured in RPMI 1640 medium (HyClone, San Angelo, TX, USA), supplemented with 10% Fetal Bovine Serum (FBS), 100 U/mL penicillin, 100 µg/mL streptomycin at 37 °C and 5% CO_2_ in a incubator.

#### MTT assay

Cells were plated in a 96-well plate at a density of 5× 10^3^ cells/well and then treated with samples at various concentrations (0.l, 0.2, 0.3, 0.4, 0.5 and 0.6 mg/mL). PBS (for PPs) or cultures without polyphenols (for biotransformation extract, respectively) were used the blank. 48 h later, 20 µL of MTT solution (5 mg/mL in PBS) was added to each well and placed in an incubator for 4 h. Then, the supernatant was removed, and the formazone crystals were dissolved using DMSO. The absorbance was then measured using a microplate reader at a wavelength of 490 nm. The results were expressed as a percentage (%) of inhibition rate calculated with the following equation:

\begin{eqnarray*}\text{Inhibition rate}(\text{%})=(1-{A}_{1}/{A}_{0})\times 100 \end{eqnarray*}

where *A*_0_ is the absorbance without sample; and *A*_1_ is the absorbance with sample. The efficient concentration of samples that inhibited 50% of cells (IC_50_) was calculated and expressed as µg/mL.

## UPLC-ESI-Q-TOF-MS

The samples were analyzed using UPLC-ESI-Q-TOF-MS (Agilent 6520 Accurate Mass Q-TOF/MS; Agilent, Santa Clara, CA, USA) in positive ion mode. Ions were generated using an electrospray ion source. The UPLC apparatus used (Waters Acquity; Waters, Bilford, MA, USA) consists of a binary pump, a quaternary pump, a solvent degasser, an autosampler and a thermostat column compartment. Samples were carried on a C18 column (Waters Acquity BEH C18, 50 mm × 2.1mm, 1.7 µm; Waters, Bilford, MA, USA) at a column temperature of 40 °C. The binary mobile phase consists of water with 0.1% formic acid (A) and acetonitrile with 0.1% formic acid (B). The 13-min-long gradient was as follows: 0–2 min, 5–10% B linear, 2–4 min, 10–30% B linear, 4–8 min, 30–80% B linear, 8–10 min, 80–100% B linear, 10–11 min, 100% B isocratic, 11–12 min, 100–5% B linear, followed by 1 min of re-equilibration of the column before the next run. The flow rate was maintained at 0.25 mL/min.

The nitrogen pressure and flow rate on the nebulizer were 25 psi and 10 L.min^−1^, respectively, with a drying gas temperature of 330 °C. The capillary voltage was 4 kV. The scan range was set at m/z 50–1,500. The fragmentor voltage was fixed at 100 V. UPLC-ESI-TOF-MS data were acquired under positive ion mode using Mass Hunter (Agilent) software.

### Statistical analysis

All tests were performed in triplicate and the results were presented as mean ± standard deviation (SD). Differences between mean values were compared by the Tukey *post hoc* test using the SPSS 18 software. Differences with  *p* < 0.05 were considered significant, *p* < 0.01 very significantly.

## Results and Discussion

### Polyphenols content

**Figure 1:**
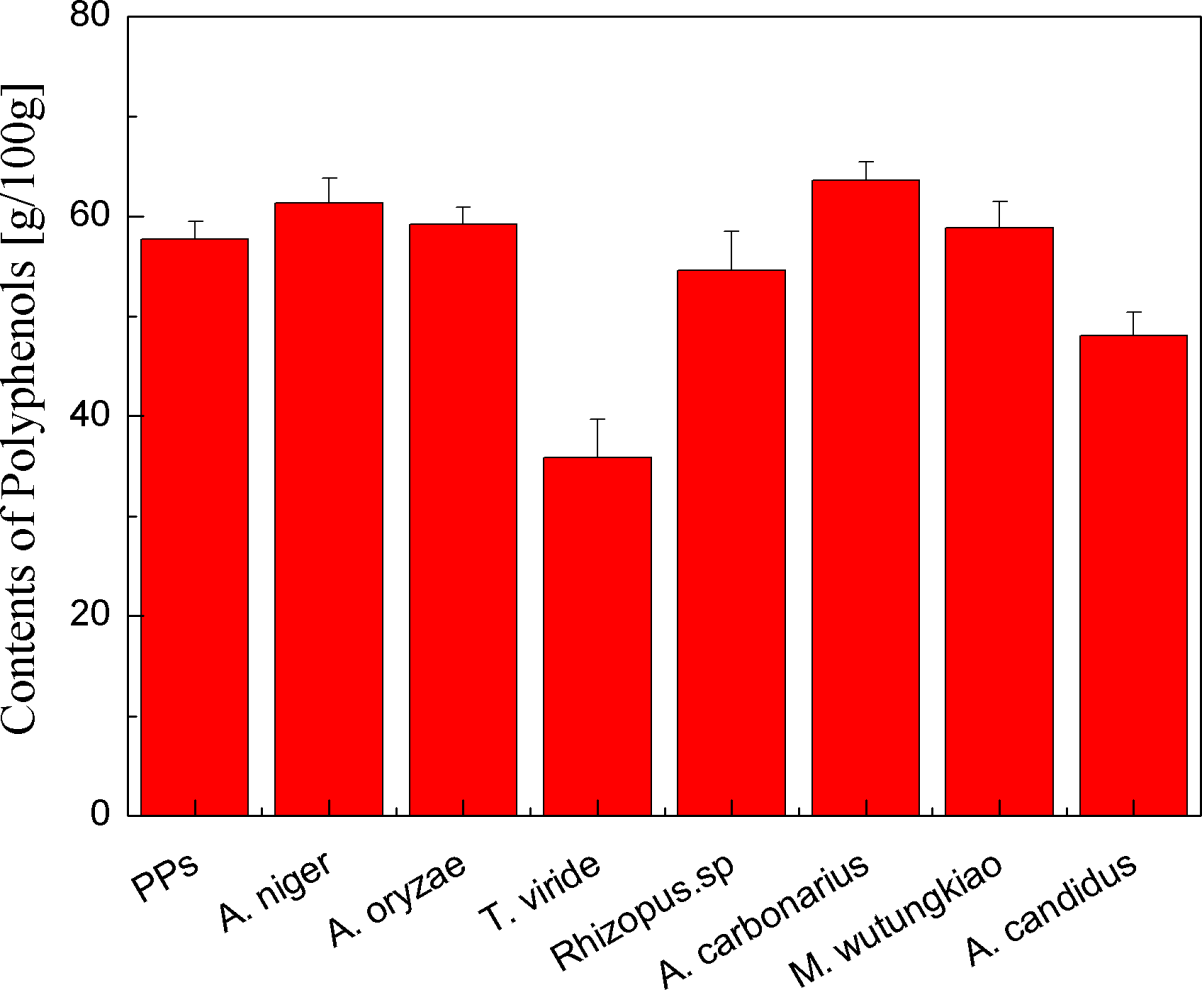
Polyphenols contents of the pine polyphenols (PPs) and seven biotransformation extracts. Each value represents mean ± SD (*n* = 3).

Polyphenols contents of the PPs and seven biotransformation extracts were investigated and illustrated in Fig. 1. After 48 h of biotransformation, Polyphenols contents of BAN and BAC increased. Fungus *Rizhopus oryzae* fermented rice bran increase polyphenols content by more than two times (Schmidt et al., 2014). *Aspergillus niger, Aspergillus niveus* and *Aspergillus awamori* fermented defatted soybean flour significantly increase in polyphenols content and fungi *β*-glucosidases might have hydrolysis *β*-glucoside linkages, mobilizing polyphenols to react with the Folin-Ciocalteau reagent (Georgetti et al., 2009). The tea polyphenols contents of pu-erh tea (*Camellia assamica)* in a fermentation solid system with *Aspergillus niger* and *Aspergillus fumigatu* are changed with fermentation time (Qin et al., 2012). Therefore, fungus *Aspergillus niger*, *Aspergillus carbonarius* and *Mucor wutungkiao* increase polyphenols content.

### Yield of polyphenols in biotransformation extract

**Figure 2:**
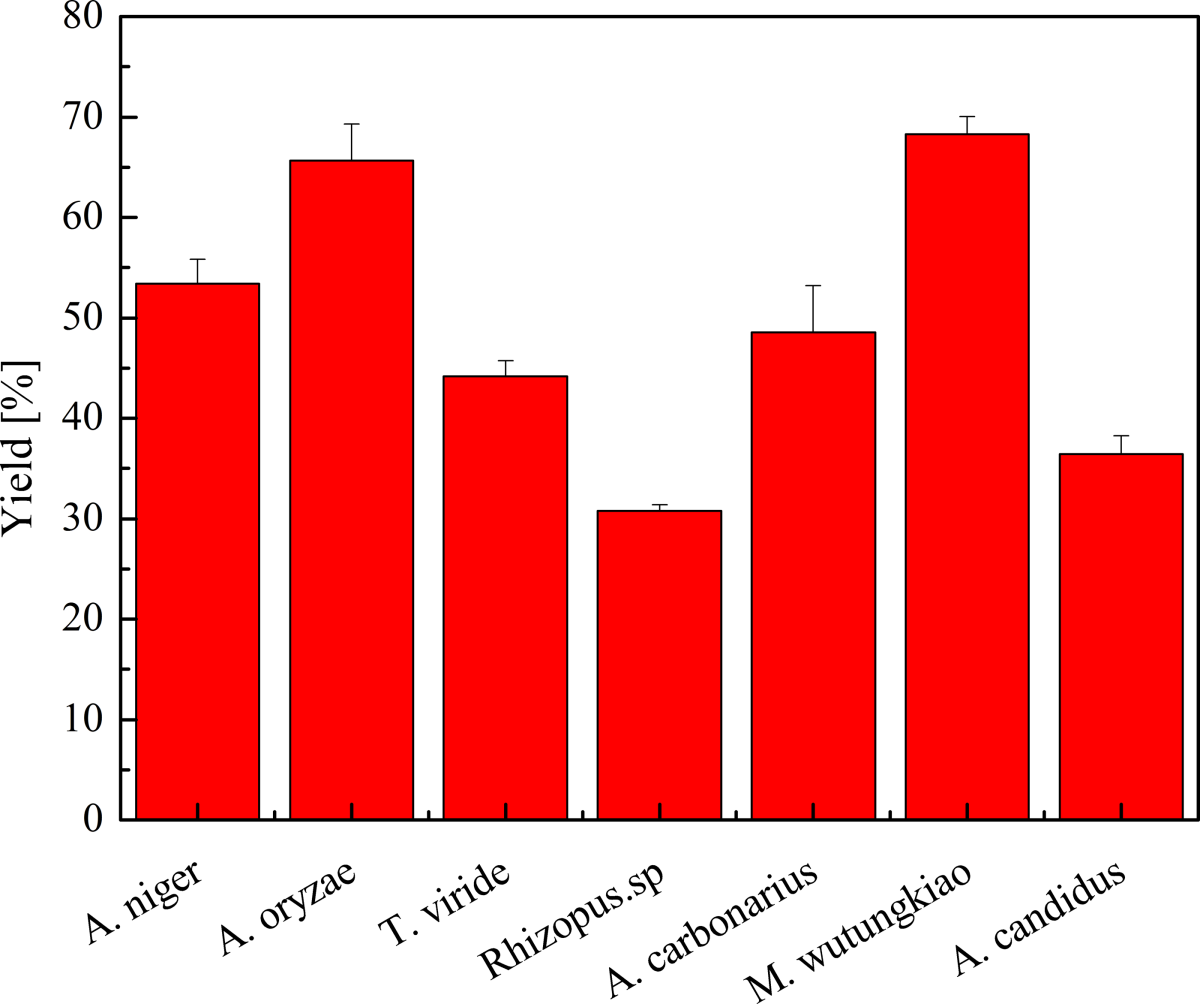
Yield of polyphenols in biotransformation extract. Each value represents mean ± SD (*n* = 3).

Yield of polyphenols in seven biotransformation extracts was investigated and shown in Fig. 2. After the process of biotransformation and chromatographic separation, BMW exhibited the highest yield of polyphenols, followed by BAO, BAN and BMW. *Bifidobacterium animalis* B94 significantly decrease the content of EGC in the medium (López de Lacey et al., 2014). *Saccharomyces cerevisiae* irreversible adsorbs grape and wine tannins (Mekoue Nguela et al., 2015). Fungi may also have irreversible adsorption of polyphenols, partly resulting in the decrease the yield of polyphenols.

### Antioxidant activity

**Figure 3:**
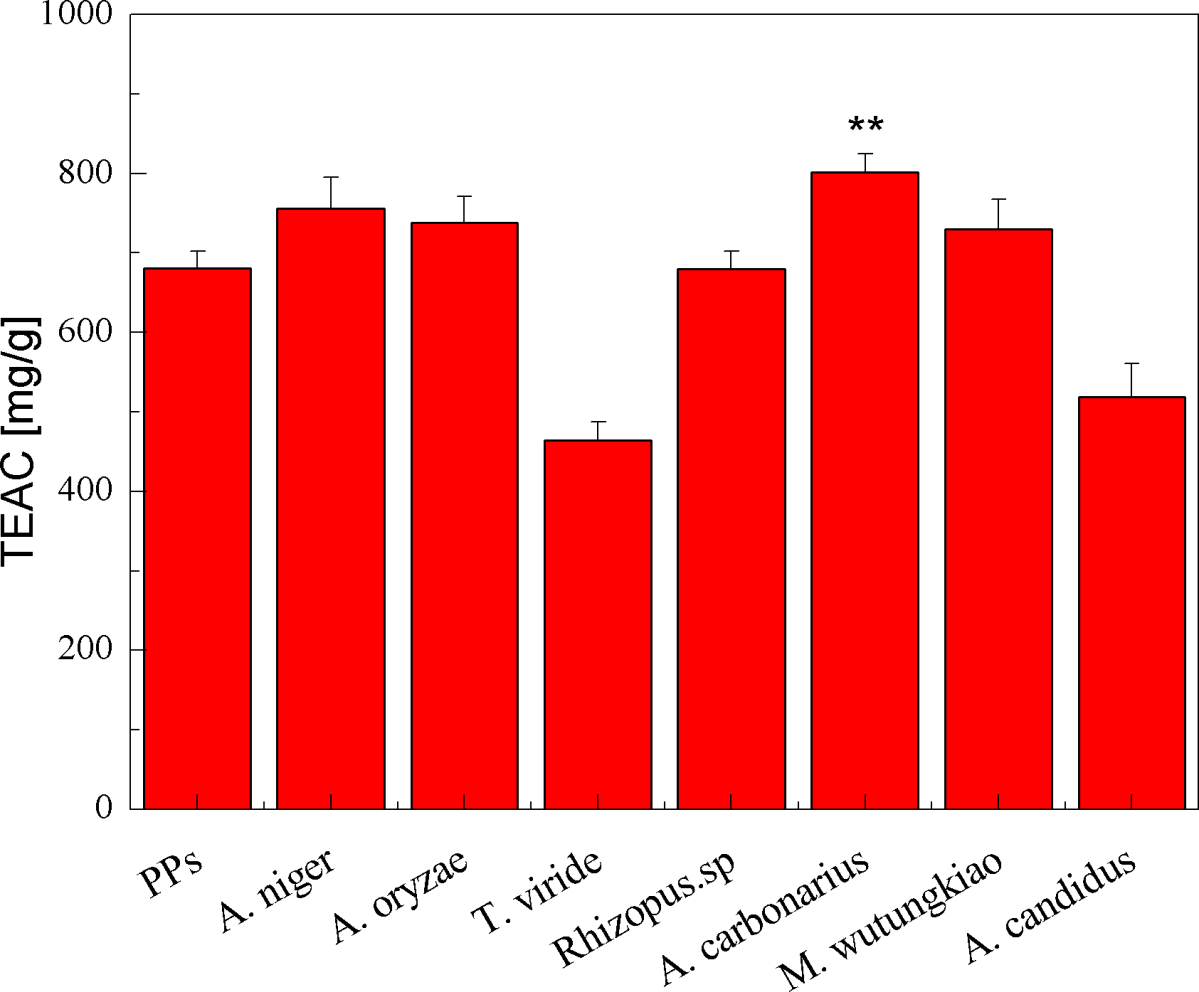
TEAC of the pine polyphenols (PPs) and seven biotransformation extracts. Each value represents mean ± SD (*n* = 3). * represent value significantly different from the PPs (*p* < 0.05); ** represent value very significantly different from the PPs (*p* < 0.01).

The TEAC of the PPs and seven biotransformation extracts was investigated and illustrated in Fig. 3. The total polyphenols assay using the Folin-Ciocalteu reagent, DPPH radical and ABTS^⋅+^ radical scavenging activity assays is considered under the electron transfer (ET) mechanism. ET-based method involves two components in the reaction mixture, antioxidants and oxidant. Oxidant abstracts an electron from the antioxidant, resulting in the color changes of the oxidant. The degree of the color change is proportionate to the antioxidant concentration (Karadag, Ozcelik & Saner, 2009). BAC showed the highest TEAC (*p* < 0.01). Compared with the PPs, BAN, BAO and BMW also showed higher but not significant TEAC.

**Figure 4:**
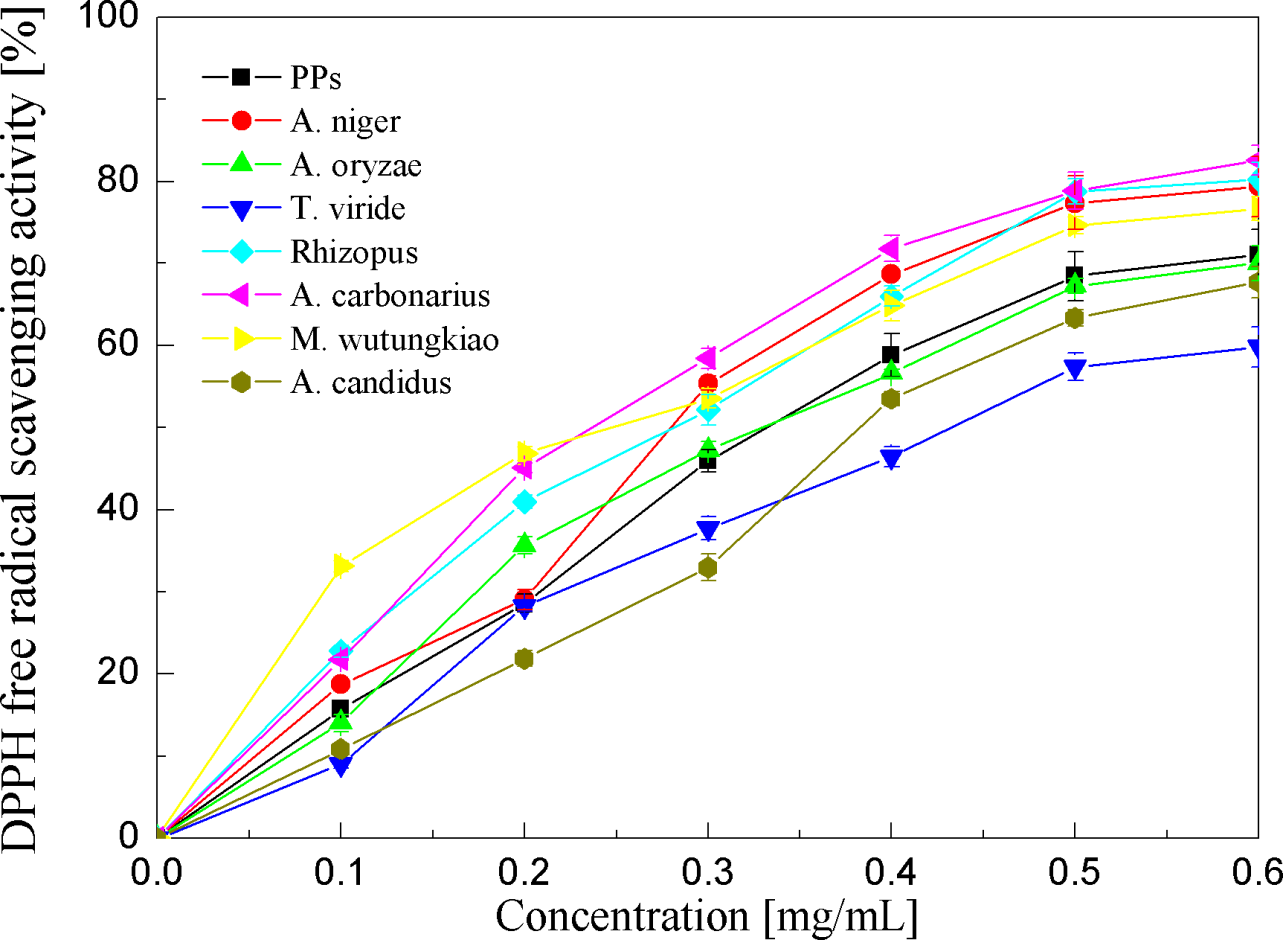
DPPH free radical scavenging activities of the pine polyphenols (PPs) and seven biotransformation extracts. Each value represents mean ± SD (*n* = 3).

DPPH free radical scavenging activities of the PPs and seven biotransformation extracts were investigated and shown in Fig. 4 and Table 1. DPPH free radicals are hydrophobic, nonphysical and long-lived organic nitrogen radicals under a deep purple color. The PPs and seven biotransformation extracts scavenge DPPH free radicals in a dose-dependent manner at concentrations of 0.1–0.6 mg/mL. It is found that the PPs and seven biotransformation extracts all exhibit DPPH radical scavenging activity, and BMW, BAC, BRS and BAN show higher DPPH free radical scavenging activity than the PPs (*p* < 0.01).

**Table 1:** EC_50_ of antioxidant activities of the pine polyphenols (PPs) and seven biotransformation extracts.

Fungi	EC_50_ (µg/mL)			RP_0.5_ (µg/mL)
	DPPH^⋅^	O${}_{2}^{-\cdot }$	OH^⋅^	
PPs	324.0 ± 13.52	117.1 ± 6.13	519.9 ± 25.91	363.2 ± 5.34
*A. niger*	265.7 ± 8.33^b^	84.2 ± 4.04^a^	310.4 ± 9.72^b^	232.2 ± 9.12^b^
*A. oryzae*	322.9 ± 10.41	122.3 ± 8.12	628.8 ± 36.94	231.6 ± 7.53^b^
*T. viride*	425.9 ± 15.84	292.5 ± 23.32	435.0 ± 26.94	297.7 ± 16.21^b^
*Rhizopus.sp*	243.5 ± 6.16^b^	97.0 ± 2.84	649.0 ± 32.65	594.1 ± 17.54
*A. carbonarius*	226.6 ± 4.27^b^	72.7 ± 3.06^b^	367.7 ± 19.02^b^	271.3 ± 10.67^b^
*M. wutungkiao*	213.3 ± 4.43^b^	85.5 ± 3.97^a^	633.9 ± 36.73	248.2 ± 13.42^b^
*A. candidus*	390.5 ± 13.44	95.7 ± 3.02	381.5 ± 15.91^b^	388.6 ± 5.29

**Notes.**

Each value represents mean ± SD (*n* = 3).

a Represent value significantly different from the PPs (*p* < 0.05).

b Represent value very significantly different from the PPs (*p* < 0.01).

EC_50_ (µg/mL) values are calculated from the regression lines using six different concentrations (50–1,200 µg/mL) in triplicate and their data are presented as 50% scavenging activity.

RP_0.5_ values are presented by the sample concentrations at 0.5 of absorbance value at 700 nm.

**Figure 5:**
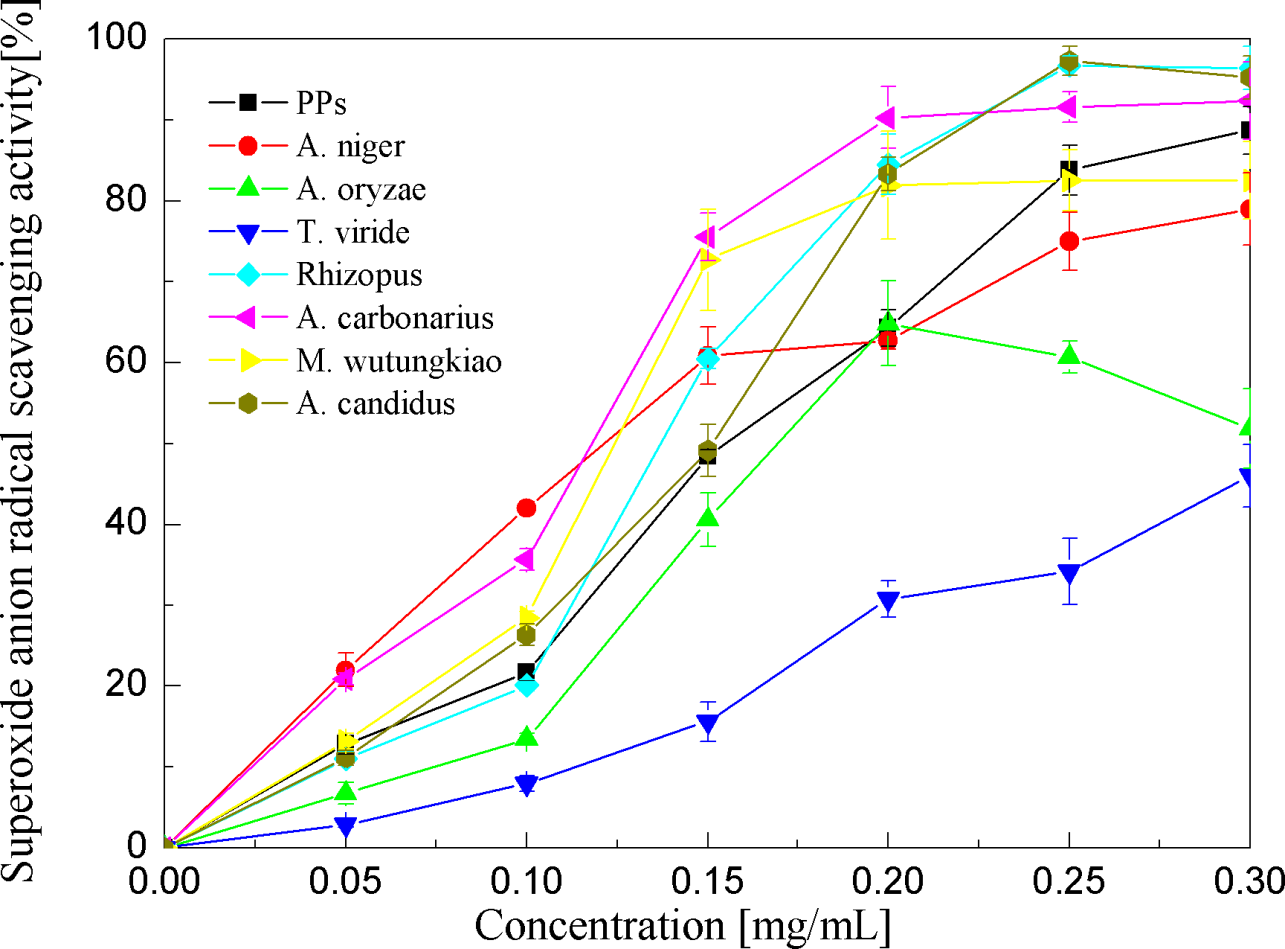
Superoxide anion radical scavenging activities of the pine polyphenols (PPs) and seven biotransformation extracts. Each value represents mean ± SD (*n* = 3).

Superoxide anion radical scavenging activities of the PPs and seven biotransformation extracts were investigated and depicted in Fig. 5 and Table 1. The PPs and seven biotransformation extracts scavenge superoxide anion radicals in a dose-dependent manner at concentrations of 0.05–0.3 mg/mL. Superoxide anion is a reduced form of molecular oxygen by receiving one electron. Flavonoids, including epicatechin, myricetin, rutin, catechin, epigallocatechin, quercetin, etc, can scavenge superoxide anions (Hort et al., 2008). The BAC showed the highest superoxide anion radical scavenging activity (*p* < 0.01), followed by BAN and BMW (*p* < 0.05).

**Figure 6:**
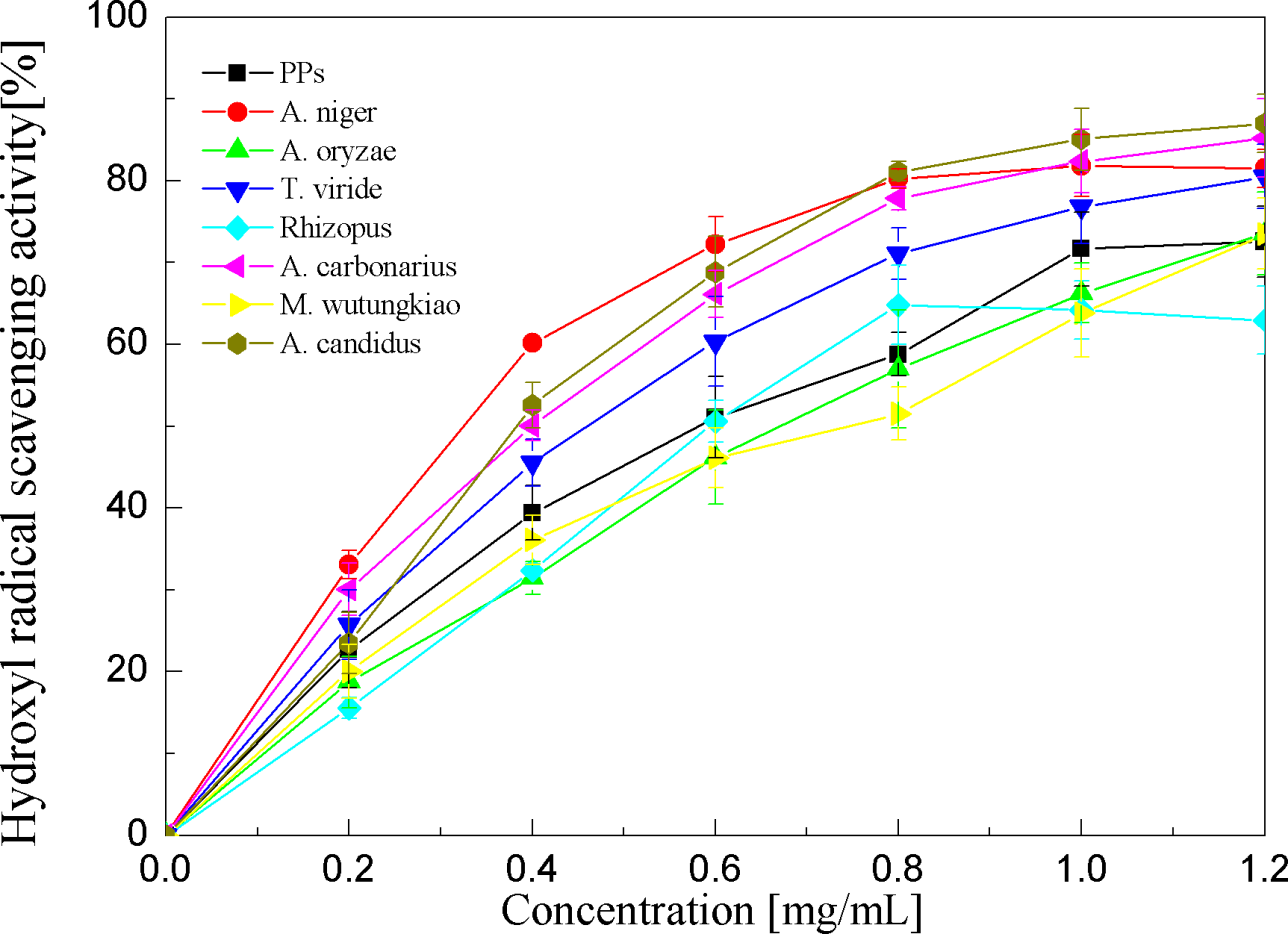
Hydroxyl radical scavenging activities of the pine polyphenols (PPs) and seven biotransformation extracts. Each value represents mean ± SD (*n* = 3).

Hydroxyl radical scavenging activities of the PPs and seven biotransformation extracts were investigated and depicted in Fig. 6 and Table 1. The PPs and seven biotransformation extracts scavenge hydroxyl radicals in a dose dependent manner at concentrations of 0.2–1.2 mg/mL. Hydroxyl radical is the most reactive free radical known and can react with every living organism. These short-lived species can hydroxylate DNA, proteins, and lipids. The BAN showed the highest hydroxyl radical scavenging activity (*p* < 0.01), followed by BAC and BACS (*p* < 0.01).

**Figure 7:**
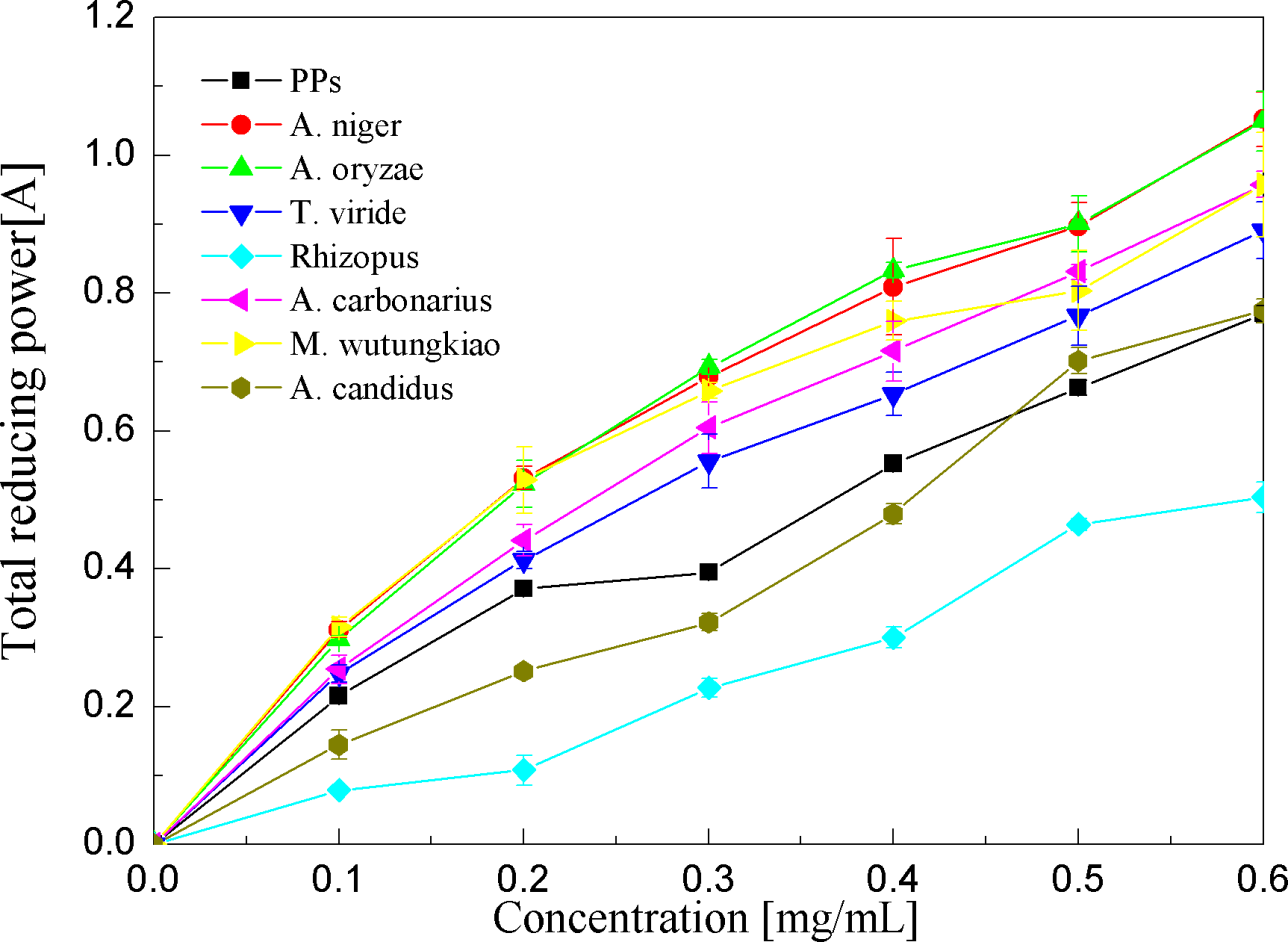
Reducing power assays of the pine polyphenols (PPs) and seven biotransformation extracts. Each value represents mean ± SD (*n* = 3).

Reducing power assays of the PPs and seven biotransformation extracts were investigated and illustrated in Fig. 7 and Table 1. The reducing power of the antioxidant was measured by the transformation of the Fe^3+^/ferricyanide complex into the ferrous form (Amarowicz et al., 2004). Reducing power assays of the PPs and seven biotransformation extracts are in a dose-dependent manner at concentrations of 0.1–0.6 mg/ml. the BAO showed the highest reducing power (*p* < 0.01), followed by BAN, BMW, BAC and BTV (*p* < 0.01).

Microbial fermentation can strengthen the food antioxidant activity. The biotransformation of green tea polyphenols by *Bifidobacterium animalis ssp. Lactis* increases ABTS^⋅+^ radical scavenging activity (López de Lacey et al., 2014). The ability of biotransformation to improve antioxidant activity is caused by an increase in the amount of polyphenols and flavonoids (Hur et al., 2014). Litchi pericarps extracted by *Aspergillus awamori* biotransformation enhance DPPH radical scavenging activity, and some new compounds such as catechin and quercetin are found, which could account for the enhanced antioxidant activity (Lin et al., 2012a; Lin et al., 2012b). Green tea (*Camellia sinensis*) and yerba mate (*Ilex paraguariensis*) after the enzymatic biotransformation reaction catalysed by the *Paecilomyces variotii* tannase increase DPPH free radical scavenging activity due to the hydrolysis of EGCG forming EGC and GA by tannase (Macedo et al., 2011). The biotransformation of xanthohumol by *Aspergillus ochraceus* forms a main transformation product and three minor metabolites; the major biotransformation product shows 8.6-fold stronger oxidation resistance than xanthohumol and 2.3-fold than ascorbic acid in DPPH free radical scavenging activity (Tronina et al., 2014).

### Antitumor activity

The cytotoxicities of the PPs and seven biotransformation extracts against LoVo, HeLa-60 and BxPC-3 cell lines were evaluated. The PPs and seven biotransformation extracts inhibit tumor cells proliferation in a dose-dependent manner at concentrations of 0.1–0.6 mg/mL. The IC_50_ values were calculated and depicted in Table 2. The BMW is active against LoVo (*p* < 0.05), and other biotransformation extracts have no significant differences compared to the PPs. Aspergillus is a class of naturally occurring microorganisms with a high value, commonly used in food, pharmaceuticals and cosmetics. *Aspergillus niger* can transform rutin to antiproliferative quercetin-3-glucosides (You, Ahe & Ji, 2010). *Aspergillus awamori* biotransformation litchi pericarps can get nigragillin and dihydrophaseic acid, which exhibits DPPH radical scavenging activity, hydroxyl radical scavenging activity, lipid peroxidation inhibition effect, DNA protection effect and antiproliferative activity against hepatoma (HepG2) and cervical cells (HeLa) (Lin et al., 2012b). The *Aspergillus oryzae*-mediated biotransformation of soybean isoflavones generates hydroxyflavones, 8-hydroxydaidzein, hydroxygenistein, and hydroxyglycitein, suggesting that biotransformation has the potential to improve the nutritional properties of soy-based food (Lee et al., 2014).

**Table 2:** IC_50_ of antitumor activities of the pine polyphenols (PPs) and seven biotransformation extracts.

Fungi	IC_50_ (µg/mL)		
	LoVo	Hela60	BxPC-3
PPs	312.5 ± 15.33	348.0 ± 16.71	348.8 ± 15.91
*A. niger*	306.9 ± 13.82	311.8 ± 12.82	297.0 ± 14.92
*A. oryzae*	301.6 ± 18.85	323.1 ± 16.14	370.8 ± 18.55
*T. viride*	474.2 ± 28.42	522.5 ± 29.53	>600
*Rhizopus.sp*	303.3 ± 13.91	558.4 ± 24.26	>600
*A. carbonarius*	285.0 ± 8.53	322.5 ± 14.64	452.1 ± 22.76
*M. wutungkiao*	258.8 ± 10.81^a^	371.1 ± 19.78	363.9 ± 16.54
*A. candidus*	346.1 ± 16.32	>600	488.0 ± 21.93

**Notes.**

Each value represents mean ± SD (*n* = 3).

a Represent value significantly different from the PPs (*p* < 0.05).

IC_50_ (µg/mL) values are calculated from the regression lines using six different concentrations (100–600 µg/mL) in triplicate and their data are presented as 50% inhibitory activity.

### Correlation analyses of polyphenols, antioxidant and antitumor

Correlation coefficients of polyphenols contents, antioxidant and antitumor of the PPs and seven biotransformation extracts were investigated and the results were shown in Table 3. A highly linear correlation between polyphenols contents and antitumor (*R*^2^ = 0.8021) and TEAC (*R*^2^ = 0.9130), DPPH free radical scavenging activity (*R*^2^ = 0.6843), and superoxide anion radical scavenging activity (*R*^2^ = 0.7313) imply that polyphenols are the main components contributing to antioxidant and antitumor activities. The results can be compared with the results of Heo et al. (2014), where an investigation *in vitro* reveals that the extracts of the indigo plant is effective in suppressing the proliferation of cancer cells, although the results vary depending on the different parts of indigo, the concentrations of solvent and extracts, the antioxidant activities and the types of cancers. Thus, different biotransformation extracts have different antioxidant and antitumor activities, and their antioxidant and antitumor activities are not only related to polyphenols contents, but also related to types of free radicals and tumors.

**Table 3:** Correlation coefficients between polyphenols contents and antioxidant, antitumor activities of the pine polyphenols (PPs) and seven biotransformation extracts.

*R*^2^	Antioxidant					Antitumor		
	TEAC	DPPH^⋅^	O${}_{2}^{-\cdot }$	OH^⋅^	RP	LoVo	Hela60	BxPC-3
Polyphenols	0.9130^b^	0.6843^a^	0.7313^b^	0.0001	0.0555	0.8021^b^	0.5093	0.0427

**Notes.**

a Represent value significantly different (*p* < 0.05).

b Represent value very significantly different (*p* < 0.01).

### Components analysis of the pine polyphenols and the BAC

**Figure 8:**
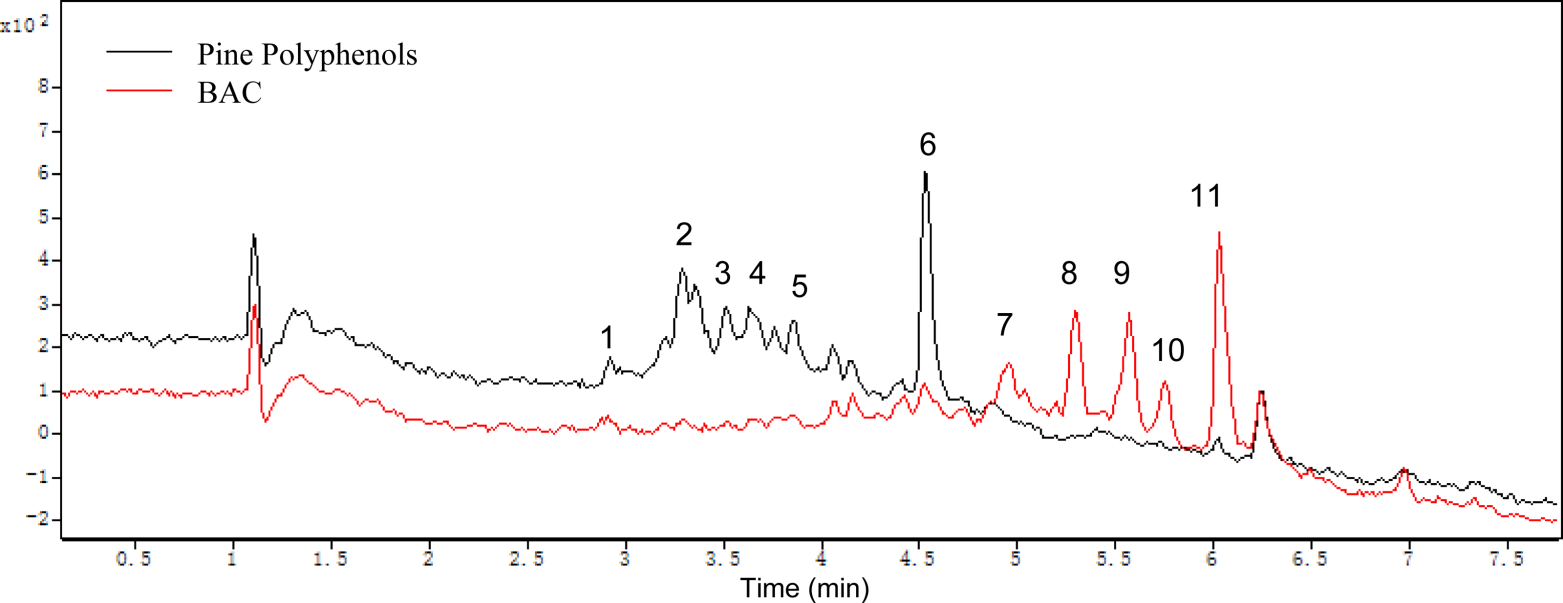
Mass spectrograms of the pine polyphenols (PPs) and the BAC.

All components of the PPs and the BAC were analyzed by HPLC-ESI-TOF-MS. Postive mode was employed in MS detection. Figure 8 shows the HPLC analysis of the PPs and the BAC with MS spectra of each target peak in the full scan mode. The MS spectrum mainly shows the ions corresponding to the protonated molecule [M + H]^+^ which can provide the molecular weight of each compound, the cleavage of the sugar unit and other easily broken unit, which can provide the neutral loss information (Delcambre & Saucier, 2012; Sang et al., 2011). Elution order by chromatographic separation, mass values (m/z), MS fragmentation combined with previously published literature analyze the components of the PPs and the BAC. The ESI/MS spectra and components fragmentation patterns were illustrated in supporting information. Peak 1 (retention time (tR) = 2.92 min) revealed a molecular ion at m/z 453 and a fragment ion at m/z 291, indicating it is a catechin derivative. The neutral loss of 162 mass units corresponded to one molecule of hexose. As a result, peak 1 is tentatively identified as catechin 3-glucoside (Delcambre & Saucier, 2012). Peak 2 (tR = 3.35 min) produced molecular ions at m/z 549 and fragments with m/z 283 and m/z 163, indicating it is a flavanone derivative. According to the literature, peak 2 has been tentatively identified as flavanone 3-rhamnosyl-glucoside (Sekkoum, Belboukhari & Cheriti, 2014). Peak 3 (tR = 3.52 min) corresponded to the molecular ion at m/z 463 and the fragment ion at m/z 317 identical with the molecular ion of isorhamnetin aglycone and the difference between both molecules corresponded to 146, corresponding to a rhamnoside moiety. Thus this compound is tentatively identified as isorhamnetin 3-O-rhamnoside (Middha et al., 2013). Peak 4 (tR = 3.64 min) had a molecular ion m/z 501 and the fragment ion m/z 355 agreed with prenylkaempferol 3-O-rhamnoside (Li, Zhang & Xiao, 1996). Peak 5 (tR = 3.85 min) showed a molecular ion m/z 547 and a major fragment ion m/z 317 for isorhamnetin 3-(butenoyl-glucoside) (Hassanean & Desoky, 1992). Peak 6 (tR = 4.54 min) revealed a molecular ion at m/z 269, indicating it is a methoxyflavonol (Kralj et al., 2013). Peak 7 (tR = 4.96 min) produced molecular ions at m/z 499 and fragments ions with 317 and 153, indicating it is a isorhamnetin derivative. According the literature, peak 7 is tentatively identified as isorhamnetin 3-glucoside (Wang et al., 2012). Peak 8 (tR = 5.31 min) corresponded to the molecular ion at m/z 293 identical with the molecular ion of a ring-opening catechin (Marfak et al., 2004). Peak 9 (tR = 5.58 min) corresponded to the molecular ion at m/z 517 and the fragment ion at m/z 287 identical with the molecular ion of kaempferol 3-diacetylrhamnoside (Delgado de la Torre, Priego-Capote & Luque de Castro, 2015). Peak 10 (tR = 5.66 min) showed a molecular ion m/z 315 for ermanin (Martinez et al., 1997). Peak 11 (tR = 6.03 min) showed a molecular ion m/z 355 and a fragment ion at m/z 315, indicating it is a prenylkaempferol (Li, Zhang & Xiao, 1996).

In this study, there are eleven polyphenols identified in the PPs and the BAC, and the results showed that the main reactions acted in the PPs by *Aspergillus carbonarius* are deglycosylation, hydroxylation, ring-opening and methylation, *etc*, which show a good agreement with the other works (Cao et al., 2015; Zafar, Ahmed & Khan, 2016).

Aspergillus, Mucor and Ttrichoderma can express and secrete polyphenol oxidase, including laccase and tyrosinase, to the outside of the cell (Balaji, Arulazhagan & Ebenezer, 2014). Polyphenol oxidase can oxidize and depredate polyphenols, and form the other polyphenols. Aspergillus also secretes glycosyl transferase, hydroxylase and methylase, which further modifies polyphenols (Hibi et al., 2016; Wallis, Hemming & Peberdy, 1999).

## Conclusions

Microbial biotransformation of the PPs can affect antioxidant and antitumor activities against human cancer cells. Microbial biotransformation is a new drug discovery with low pollution, low by-products, and low toxic effects. Fungi can be directly used for the biotransformation of polyphenols, screening for functional products. In this study, *Aspergillus niger, Aspergillus oryzae, Aspergillus carbonarius* and *Mucor wutungkiao* are appropriate biotransformation fungi, which can improve the polyphenols antioxidant and antitumor activities.

##  Supplemental Information

10.7717/peerj.3264/supp-1 Supplemental Information 1 Raw data

10.7717/peerj.3264/supp-2 Supplemental Information 2 Supporting information

## List of abbreviations

PPs: Pine polyphenols
BAN: Biotransformation extract of *Aspergillus niger*
BAO: Biotransformation extract of *Aspergillus oryzae*
BTV: Biotransformation extract of *Trichodermas viride*
BRS: Biotransformation extract of *Rhizopus.sp*
BAC: Biotransformation extract of *Aspergillus carbonarius*
BMW: Biotransformation extract of *Mucor wutungkiao*
BACS: Biotransformation extract of *Aspergillus candidus*
DPPH: 2,2-Diphenyl-1-picrylhydrazyl
MTT: 3-(4,4-Dimethylthiazol-2-yl)-2,5-diphenyltetrazolium bromide
Trolox: 6-Hydroxy-2,5,7,8-tetramethylchroman-2-carboxylic acid
ABTS: 2, 2′-Azino-bis(3-ethylbenzothiazoline-6-sulfonic acid) diammonium salt

## Additional Information and Declarations

### Data Availability

The following information was supplied regarding data availability: The raw data has been supplied as a Supplemental Dataset.
