# Supplementary material for: Comparison in antioxidant and antitumor activities of pine polyphenols and its seven biotransformation extracts by fungi
Source: PeerJ. 2017 May 23;5:e3264. doi: 10.7717/peerj.3264 (PMC5444373; doi:10.7717/peerj.3264)
Supplement: Supplemental Information 2 [file peerj-05-3264-s002.doc]

Supporting information

The MS information of all components of the pine polyphenols (PPs) and the BAC are listed.

Peak 1 (retention time (tR) = 2.92 min)

Peak 2 (tR = 3.35 min)

Peak 3 (tR = 3.52 min)

Peak 4 (tR = 3.64 min)

Peak 5 (tR = 3.85 min)

Peak 6 (tR = 4.54 min)

Peak 7 (tR = 4.96 min)

Peak 8 (tR = 5.31 min)

Peak 9 (tR = 5.58 min)

Peak 10 (tR = 5.66 min)

Peak 11 (tR = 6.03 min)
